# Development of human cell biosensor system for genotoxicity detection based on DNA damage-induced gene expression

**DOI:** 10.2478/v10019-010-0010-3

**Published:** 2010-03-18

**Authors:** Valerija Zager, Maja Cemazar, Irena Hreljac, Tamara T. Lah, Gregor Sersa, Metka Filipic

**Affiliations:** 1 Institute of Oncology Ljubljana, Department of Radiotherapy and Department of Experimental Oncology, Ljubljana, Slovenia; 2 National Institute of Biology, Department for Genetic Toxicology and Cancer Biology, Ljubljana, Slovenia

**Keywords:** HepG2 cells, biosensor system, green fluorescent protein, reporter gene assay, genotoxicity, p21 promoter

## Abstract

**Background:**

Human exposure to genotoxic agents in the environment and everyday life represents a serious health threat. Fast and reliable assessment of genotoxicity of chemicals is of main importance in the fields of new chemicals and drug development as well as in environmental monitoring. The tumor suppressor gene *p21*, the major downstream target gene of activated *p53* which is responsible for cell cycle arrest following DNA damage, has been shown to be specifically up-regulated by genotoxic carcinogens. The aim of our study was to develop a human cell-based biosensor system for simple and fast detection of genotoxic agents.

**Methods:**

Metabolically active HepG2 human hepatoma cells were transfected with plasmid encoding Enhanced Green Fluorescent Protein (EGFP) under the control of the *p21* promoter (p21HepG2GFP). DNA damage was induced by genotoxic agents with known mechanisms of action. The increase in fluorescence intensity, due to *p21* mediated EGFP expression, was measured with a fluorescence microplate reader. The viability of treated cells was determined by the colorimetric MTS assay.

**Results:**

The directly acting alkylating agent methylmethane sulphonate (MMS) showed significant increase in EGFP production after 48 h at 20 μg/mL. The indirectly acting carcinogen benzo(a)pyren (BaP) and the cross-linking agent cisplatin (CisPt) induced a dose- dependent increase in EGFP fluorescence, which was already significant at concentrations 0.13 μg/mL and 0.41 μg/mL, respectively. Vinblastine (VLB), a spindle poison that does not induce direct DNA damage, induced only a small increase in EGFP fluorescence intensity after 24 h at the lowest concentration (0.1 μg/mL), while exposure to higher concentrations was associated with significantly reduced cell viability.

**Conclusions:**

The results of our study demonstrated that this novel assay based on the stably transformed cell line p21HepG2GFP can be used as a fast and simple biosensor system for detection of genetic damage caused by chemical agents.

## Introduction

Genotoxicity data play an important role in evaluating health hazards associated with exposure of humans and living organisms to chemical substances. Genotoxicity assays are needed for screening compounds that are candidate drugs, food additives, or cosmetics to assess whether the compound of interest induces DNA damage. The methods for detecting genotoxic agents are also needed to monitor contamination of water supplies with genotoxic pollutants. In addition, genotoxicity screening should be introduced to monitor environmental pollution through industrial and municipal waste disposal. Regulatory requirements for genotoxicity testing of chemicals and products such as pharmaceuticals, pesticides, food additives, and cosmetics rely on a battery of genotoxicity tests, which generally consist of an *in vitro* test for gene mutations in bacteria and mammalian cells, an *in vitro* test for chromosomal damage and an *in vivo* test for chromosomal damage in rodent hematopoetic cells.^1^ However these same methods are unsatisfactory for rapid screening for several reasons: testing can take many weeks, when it is desirable to obtain genotoxic data in a shorter time frame, or large quantities of a tested compound are needed, when only limited quantities are available, such as during drug development or in environmental monitoring when concentrated samples are tested. Here we have developed a method suitable for primary genotoxicity screening.

Genotoxic agents cause different types of damage to the DNA molecule. To counteract the consequences of DNA damage, cells have evolved complex defense mechanisms resulting in cell cycle arrest, DNA damage repair and apoptosis, which positively contribute to genomic stability. In bacteria, DNA damage or inhibition of its replication invokes a well-characterized SOS response with the induction of about 20 different genes.^2^ An even larger number of genes are involved in the cellular response to DNA damage in yeasts^3^, and in mammalian cells.^4^ Alteration in expression of these genes can be used as a surrogate for early detection and quantification of DNA damage caused by genotoxic agents. Reporter gene expression systems that measure changes in expression of DNA damage response-associated genes as the markers of DNA damage have been shown to be suitable as high-throughput screens for genotoxicity. The most widely used are bacterial systems in which genotoxic effects are identified based on the changes in expression of SOS response genes.^5^–^6^ Recently, yeast *Saccharomyces cerevisiae* DNA reporter assays in which the *RAD54* promoter is fused to green fluorescent protein (GFP)^7^ and *RAD51* promoter fused to *Renilla* luciferase^8^, have been developed.

In mammalian cells, the most prominent pathway of cellular response to DNA damage is activation of the tumor suppressor and transcription factor p53 through phosphorylation by DNA damage-responsive kinases.^9^ Activated p53 then induces the expression of genes involved in DNA repair, cell cycle arrest, or apoptosis.^10^ The cyclin-dependent kinase 1A (CDKN1A) inhibitor *p21* (*Waf1/ Cip1*) is the major downstream target gene of activated p53 and is responsible for causing cell cycle arrest following DNA damage.^11^ The activated p53 protein directly stimulates expression of *p21* which, through its negative effect on various CDKs, inhibits both the G1 to S and the G2 to mitosis transition.^12^ In addition, by binding to the proliferating cell nuclear antigen (PCNA), p21 interferes with PCNA-dependent DNA polymerase activity, thereby inhibiting DNA replication and modulating various PCNA-dependent DNA repair processes.^13^ Up-regulation of *p21*expression upon exposure to irradiation or genotoxic chemicals has been reported in several *in vitro* and *in vivo* studies.^14^–^17^

Here we describe a new genotoxicity test system based on a *p21*-dependent GFP reporter gene assay with stably transformed human hepatoma HepG2 cells. The HepG2 cells were chosen because of their human origin and their retained activities of xenobiotic-metabolizing enzymes, which make them a better model for reflecting the processes in an intact liver than other *in vitro* test systems.^18^ In addition, HepG2 cells express wild-type tumor suppressor p53^19^, making them an appropriate model for development of the test system based on the p53-mediated DNA damage response. The results showed that this test could be used for a high throughput screening for genotoxic agents.

## Materials and methods

### Chemicals and reagents

Methyl methane sulphonate (MMS), benzo[a] pyrene (BaP) and dimethyl sulphoxide (DMSO) were purchased from Sigma, St. Louis, USA). Cisplatin (CisPt) was obtained from Medac, Hamburg, Germany, and vinblastine sulphate (VLB) from Lilly France S.A., Fagersheim, France.

### Cell line

The human hepatoma HepG2 cell line was obtained from ECACC (Wiltshire, UK), and was grown in minimum essential medium (MEM, advanced, GIBCO, Invitrogen, Paisley, UK) without phenol red supplemented with 10% heat inactivated fetal calf serum (FCS, SIGMA, St. Louis, MO, USA). Cells were routinely subcultured twice per week and were maintained in a humidified atmosphere with 5% CO_2_ at 37°C.

### Construction of plasmids

The plasmid pEGFP-N1, encoding Enhanced Green Fluorescent Protein (EGFP) controlled by the CMV promoter (Clontech, Basingstoke, UK) was used as a source of the coding sequence of the EGFP gene. The source of the coding sequence of the *p21* promoter was the WWP-LUC plasmid, which was a gift from Prof. Bert Vogelstein (Johns Hopkins Oncology Center, Baltimore, Maryland, USA). The construction of a recombinant vector containing the *p21* promoter reporter cassette and EGFP was done in several steps using the Clontech pEGFP-N1 plasmid as a backbone and standard molecular biology techniques of restriction and ligation. In addition, the gene for neomycin resistance was included into the plasmid, which enabled the isolation of HepG2 cells with stable expression of the reporter gene under pressure of Geneticin^®^ (neomycin, GIBCO). The constructed plasmid pp21-EGFP was cloned into *E. coli* (strain DH5α, Invitrogen, UK), and isolated using the Qiagen Maxi Endo-Free kit (Qiagen, Hilden, Germany), according to the manufacturer’s instructions. Purified plasmid DNA was subjected to quality control and quantity determinations, performed by agarose gel electrophoresis and by means of spectrophotometry.

### Transfection of HepG2 cell line

The HepG2 cells were transfected with the pp21-EGFP plasmid using electroporation as described.^20^ 40 μl of cell suspension (2.5 ×10^7^ cells/ml) were mixed with 10 μg of plasmid DNA and placed between two flat parallel stainless steel electrodes with a 2-mm gap and subjected to 8 square-wave shaped electric pulses of 5 ms duration, repetition frequency 1 Hz. Different electric field intensities were tested: 400 V/cm, 600 V/cm, 700 V/cm, 800 V/cm and 1000 V/cm. The electric pulses were generated by an electroporator (GT-1, electroporator, Faculty of Electrical Engineering, University of Ljubljana, Slovenia). After exposure to electric pulses, the cells were incubated for 5 min at room temperature. Thereafter, cells were maintained in non-selective medium for 1–2 days after transfection. The selection of stably transfected clones was performed by culturing the cells in medium containing 1 mg/ml Geneticin^®^. Cultivation in the selective medium was continued for 2–3 weeks. During this period, the cells without plasmid died while the cells containing stably incorporated plasmid were able to replicate and form colonies.

Separate colonies were picked and transferred into wells of 96-well microtiter plates and cultivated under pressure of 0.5 mg/ml Geneticin^®^. After reaching a sufficient number, the cells were transferred to larger plates for further propagation to obtain a sufficient number of cells for further selection of the most responsive clones. The clones with visible morphological and/or replication changes were discharged.

### Cell treatment with model genotoxic agents and EGFP measurement

Model genotoxic agents with known mechanisms of action were used to test and validate the cell biosensor system. Stock solutions were prepared prior to testing: MMS, and CisPt were dissolved in distilled water at concentrations 50 mg/mL (454 mM) and 2 mg/mL (6.7 mM), respectively. BaP was dissolved in DMSO at a concentration 2.52 mg/mL (10 mM) and VLB in 0.9% NaCl at a concentration 1 mg/mL (1.1 mM). Further dilutions were made in cell culture media.

A suspension of exponentially growing p21HepG2GFP cells (3×10^5^ cells/mL) in minimum essential medium without phenol red with 10% fetal calf serum was distributed in 3 mL aliquots to plastic test tubes. 30 μL of test chemical of appropriate concentration (100-fold higher concentrations than final treatment concentrations) or 30 μL of vehicle for controls were added to each tube. The following final concentrations were used: MMS: 5, 10, 20, 40, 50 μg/mL; CisPt: 0.4125, 0.825, 1.65, 3.3, 6.6 μg/mL; BaP: 0.05, 0.13, 0.25, 0.5, 1.26 μg/mL, and VBL 0.1, 0.5, 1.0, 2.5, 5.0 mg/mL. For the EGFP fluorescence measurements, 100 μL aliquots from each tube were distributed to 6 wells of 96-well black microtiter plates with a clear bottom (Greiner BIO-ONE, Nuernberg, Germany). The plates were incubated at 37°C, 5% CO_2_ for 7 days, and the EGFP fluorescence was determined after 24, 48, 72, 120 and 168 h. The intensity of EGFP fluorescence was measured at 485 nm excitation and 535 nm emission wavelengths with a fluorescence microplate reader (Tecan Infinite 200). The experiments were repeated three times.

From fluorescence intensity measurements, a relative EGFP induction ratio was calculated. Fluorescence intensity of the treated cells was divided by the fluorescence intensity of control cells and normalized to the relative cells viability determined with the MTS assay.

### Determination of cell viability (MTS assay)

The cell viability was determined by the colometric (3-(4.5-dimethylthiazol-2-yl)-5-(3-carboxy-methoxyphenyl)-2-(4-sulfophenyl)-2H-tetrazolium, inner salt) MTS assay with the CellTiter 96 Aqueous One Solution Cell Proliferation Assay (Promega, Madison, USA) according to the manufacturer’s instructions. The 100 μL aliquots from each test tube of treated or control cells were distributed into 4 wells of normal 96 well microtiter plates and incubated for 24, 48, 72 120 or 168 h. For each of the 5 time point measurements, a separate microtiter plate was prepared. At the end of the incubation period with chemical agents, 20 μL of MTS solution were added to each well of 96-well microtiter plates and incubated for 2 h in a humidified atmosphere with 5% CO_2_ at 37°C. After the incubation with MTS, the microtiter plates were shaken for 30 s and the absorbance of the resulting solution was measured at 492 nm using a Labtec HT2 microplate reader (Anthos, Wals, Austria). Relative survival of cells was calculated by dividing the absorbance of the treated cells with the absorbance of the control cells. The experiments were performed in quadruplets and repeated 3-times.

### Statistical analysis

Statistical analysis was performed using SigmaStat software (Systat Software, Inc., Richmond CA). All data were first tested for normality with the Kolmorogov-Smirnov normality test. Significance tests were carried out using analysis of variance (ANOVA) and two-tailed Student’s t-test. Values of p<0.05 were considered significant. Data were presented as the arithmetic mean (AM) ± standard deviation of the mean (SD).

## Results

### Construction of reporter gene plasmid and stably transformed HepG2 cells

**FIGURE 1:**
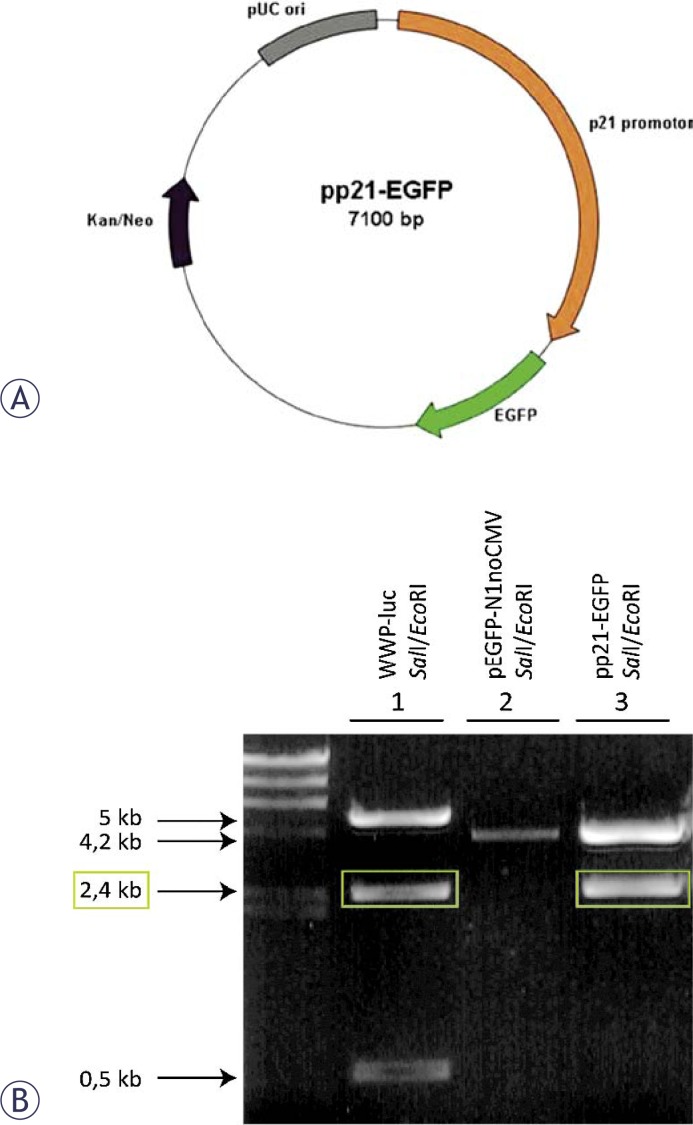
pp21-EGFP plasmid (A) and confirmation of successful construction of the plasmid (B). The identity of the plasmid was confirmed with SaII and EcoRI restriction. Sample 1 is WWP-Luc from which the p21 promoter (marked yellow) was isolated. Sample 2 is linearized pEGFP-N1 without CMV plasmid from which CMV was cut out with the same restriction enzymes to form blunt ends. Sample 3 is pp21-EGFP plasmid resulting from ligation of a 2.4 kB p21 promoter from WWP-Luc and sample 2 restricted with SaII and EcoRI.

For this genotoxicity screening system, a plasmid pp21-EGFP with the *p21* promoter inserted in front of the EGFP reporter gene was constructed (Figure 1A). Successful construction and isolation of the pp21-EGFP plasmid was confirmed with restriction analysis (Figure 1B). The pp21-EGFP plasmid was then transfected to HepG2 cells. In the final step, HepG2 cell clones expressing low basal and high inducible EGFP expression were isolated.

**FIGURE 2:**
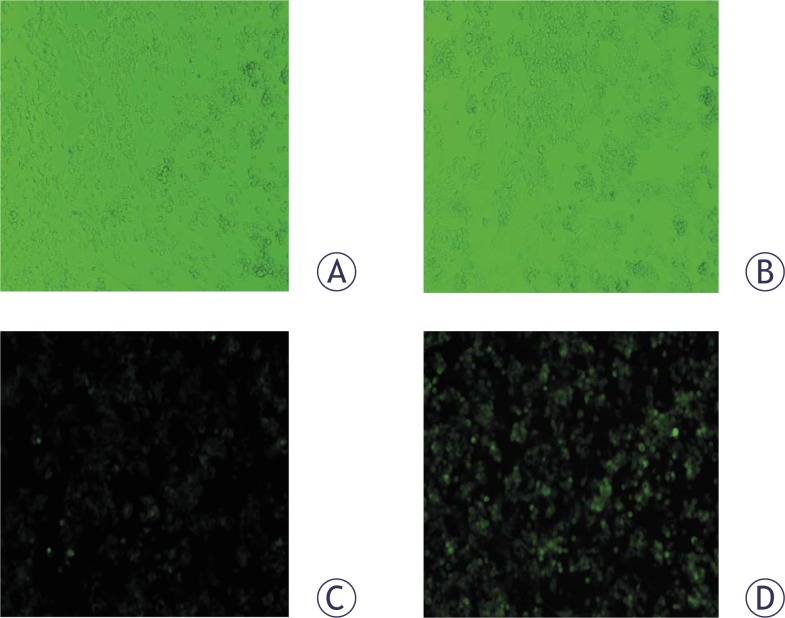
Photomicrographs of control (A, C) and p21HepG2GFP cells exposed to 50 μg/ml MMS for 48 hours (B, D). Images taken under visible light condition (A, B) and images taken fluorescence epi-illumination (C, D).

For the isolation of DNA damage-responsive clones we used MMS. After measuring the basal and MMS induced EGFP levels in 36 independent clones the one with the highest inducible and the lowest basal level of EGFP expression was selected for further propagation and characterization and for the experiments with the known model genotoxic compounds. The clone was named p21HepG2GFP. Microscopic observations of p21HepG2GFP cells demonstrated a clear increase of EGFP fluorescence intensity induced by 50 μg/mL MMS after 48 h exposure (Figure 2).

### Cell viability as an internal standard

**FIGURE 3:**
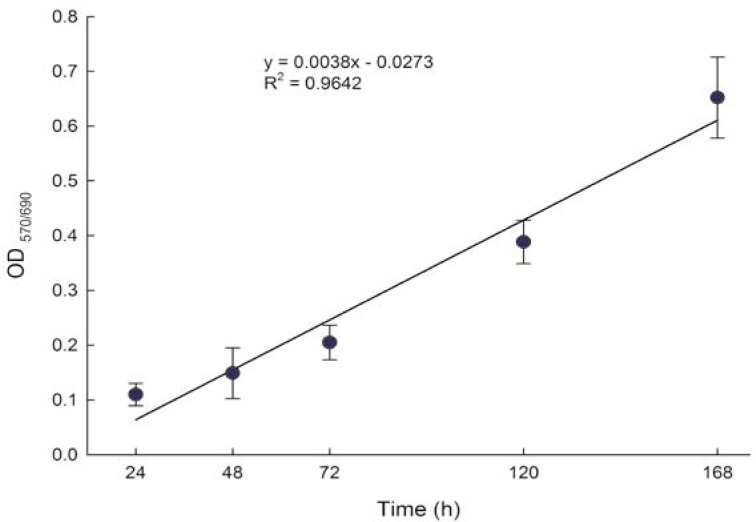
Proliferation of p21HepG2GFP cells measured with the MTS assay. 5000 cells per well were plated on 96-well microtitre plates in triplicate and incubated for 24, 48, 72, 120 and 168 h. The values represent means of four independent experiments ± SD.

Since it is known that genotoxic chemicals are toxic at certain concentrations and thus suppress cell growth during exposure which was continued for up to 7 days, it was necessary to normalize the observed level of EGFP to the number of viable cells. The induction of EGFP fluorescence was measured after 24, 48, 72, 120 and 168 h on the same population, while this was not possible for determination of cell viability, since no appropriate method that would allow for determination of cell viability without termination of cell culturing is available. The MTS assay that measures the conversion of MTS to the formazan product by dehydrogenase enzymes of the intact mitochondria of living cells correlates with the number of viable cells. We therefore used this assay to indirectly determine the relative changes in cell numbers during the exposure to tested chemicals. For each treatment, we prepared five plates for the measurement of cell viability (one plate for each time point) in parallel to the plate for EGFP fluorescence measurements. The correlation analysis of the proliferation of p21HepG2GFP cells showed that absorbance of the formed formazan product correlated to cell proliferation (r = 0.94) (Figure 3). The data also indicate that during the exponential growth phase the doubling time of the p21HepG2GFP cells is about 48 h. At each time point, the relative cell viability compared to non-treated control cells was calculated and the factor was used for normalization of the relative EGFP induction ratio to the number of viable cells. A reduction of relative cell viability by more than 30% (reduction factor 0.7) was considered as cytotoxic.

### Responses of p21HepG2GFP cells to exposure to model genotoxic agents

To demonstrate the sensitivity of this bioassay for detection of genotoxic agents, we tested several genotoxic agents with known mechanisms of action. To determine the optimal exposure conditions, a time and dose dependence of *p21*-dependent EGFP fluorescence induced by model genotoxic agents was investigated.

**FIGURE 4:**
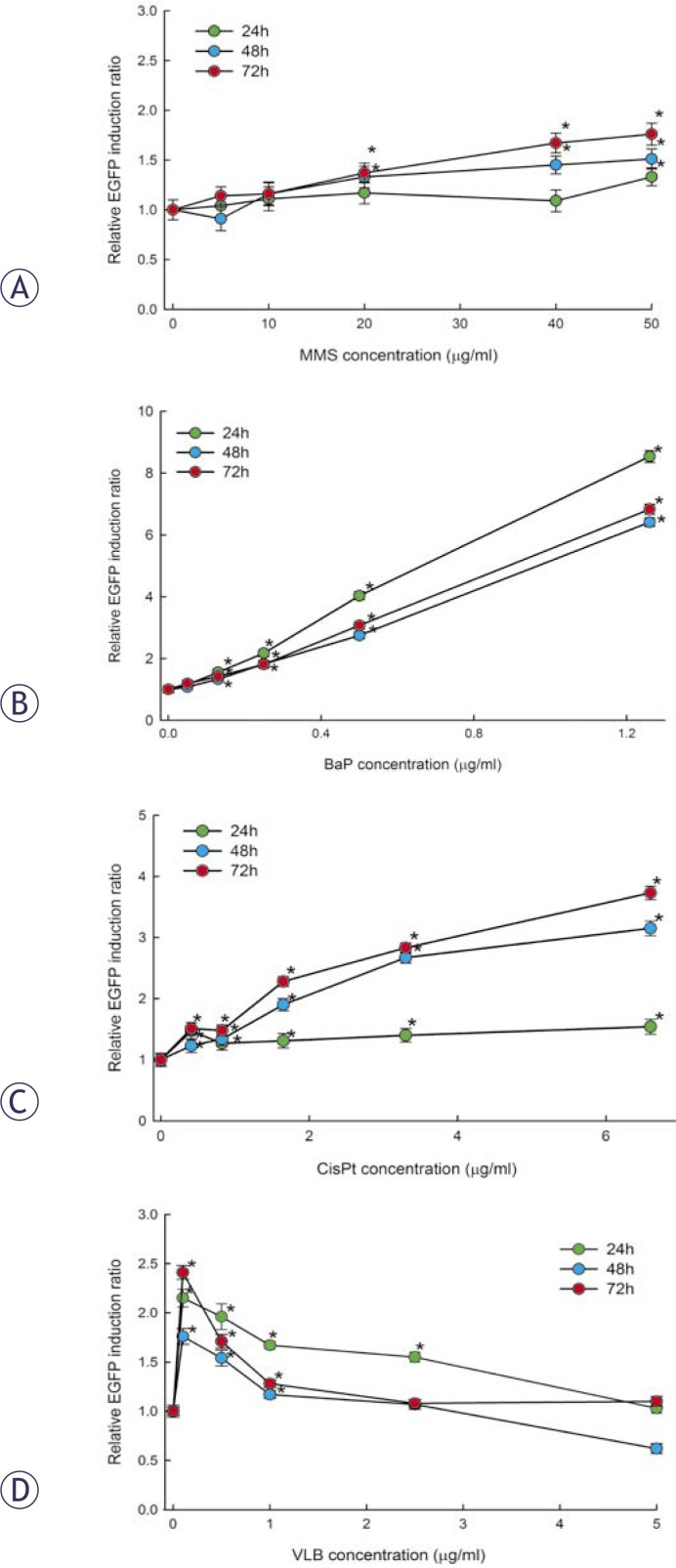
Dose- and time-dependent induction of EGFP expression in p21HepG2GFP cells treated with graded doses of MMS (A), BaP (B), CisPt (C) and VBL (D) after 24, 48 and 72 h of exposure. The response is presented as the relative EGFP induction ratio which is the ratio between EGFP fluorescence of the treated cells and the background fluorescence of control cells normalized to the relative cell viability. The values represent means of four independent experiments ± SD; *p<0.05

*Methyl methane sulphonate (MMS),* a direct acting genotoxic agent that induces alkylation of DNA bases, induced a statistically significant increase in EGFP fluorescence after 24 h to 50 μg/mL and after 48 h exposure to 20, 40 and 50 μg/mL (Figure 4A, Table 1). The MMS-induced increase of EGFP fluorescence was time- and dose-dependent, which is clearly reflected in the increasing values of relative EGFP induction ratio (Figure 4A, Table 1). After 120 and 168 h exposure, a significant increase in EGFP fluorescence associated with the increase in relative EGFP induction ratio was observed at all concentrations (Table 1). The parallel measurement of cell viability during the exposure to MMS showed that it was not significantly affected during the initial 72 h of exposure, while after 120 and 168 h it was reduced by more than 30% compared to non-treated control cells (Table 1).

*Benzo[a]pyrene* (*BaP)* is a mutagenic and carcinogenic indirectly-acting genotoxic agent which forms BaP diolepoxide (BPDE)-DNA adducts after metabolic activation. BaP induced a significant dose-dependent increase in EGFP fluorescence at all exposure times and all concentrations except the lowest one (0.05 μg/mL). However, the relative EGFP induction ratio did not increase with prolonged exposure indicating that the EGFP induction reached a plateau (Figure 4B, Table 1). BaP did not significantly reduce the cell viability during the exposure up to 72 h (Table 1), while with further exposure the viability was reduced by more than 30% at all doses of BaP (Table 1).

*Cisplatin (CisPt),* a well known chemotherapeutic, is a directly-acting genotoxic agent that induces alkylation of DNA and DNA cross-links. CisPt induced significant increase of EGFP fluorescence already after 24 h exposure at all concentrations. With further exposure, the relative EGFP induction ratio tended to increase with the time of exposure (Figure 4C, Table 1). In cells exposed to 3.3 μg/mL CisPt, the relative EGFP induction ratio increased from 1.40, determined after 24 h, to 2.83 determined after 72 h of exposure (Figure 4C, Table 1). CisPt did not reduce cell viability after 24 h of exposure. After 48 and 72 h exposure, the viability of the cells was significantly reduced at the two highest concentrations (3.3 and 6.6 μg/mL) while after 120 and 168 h exposure, CisPt reduced cell viability by more than 30% at all tested concentrations (Table 1) *Vinblastine (VLB)* is a chemotherapeutic that does not induce DNA damage but induces disturbances in cell replication due to its interference with mitotic spindle formation. This compound induced significant increase of EGFP fluorescence after 24 h exposure to all concentrations, except the highest (5.0 μg/μL). After 48 h exposure, a significant increase of EGFP fluorescence was detected at the lowest three concentrations (0.1, 0.5 and 1.0 μg/mL), while at higher concentrations and with prolonged exposure the EGFP fluorescence intensity was reduced (Figure 4D, Table 1). The viability measurements showed that VBL was highly cytotoxic. Although after 24 and 48 h exposure cell viability was not reduced by more than 30%, except at the highest concentration, after prolonged exposure it rapidly decreased. After 72 h exposure the viability was reduced by more than 40% at all concentrations and after 168 h exposure it decreased by more than 90% compared to the viability of non-treated control cells (Table 1).

## Discussion

We developed a novel microplate genotoxicity assay test system using EGFP as the reporter that enables simple and rapid detection of genotoxic agents. The assay is based on a p21HepG2GFP cell line that contain the EGFP reporter under the control of the *p21* promoter. In response to DNA damage, the transcription of the *p21* promoter is activated leading to concurrent accumulation of EGFP that is detected in intact cells with the fluorescence microplate reader.

Several reporter genotoxicity assays using mammalian cells and DNA damage responsive genes as the biomarkers of genotoxic injury have been described. Todd *et al.*^21^ were the first who exploited DNA damage responsive genes: *p53R2*, *GADD45a* and *GADD153* for construction of a chloramphenicol acetyl transferase (CAT) reporter that was stably integrated into HepG2 cells. However, there is very little data published from this assay. The *p53R2*, one of the p53 target genes that encode a subunit of ribonucleotide reductase, which is expressed mainly in response to DNA damage^22^, ^23^, has been used more recently for construction of a reporter assay with MCF7 and HepG2 cells using luciferase as the reporter gene.^24^,^25^ The growth arrest and DNA damage (*GADD*)-inducible gene family is another group of target genes regulated by p53 that are expressed in response to various environmental stresses including DNA damage. In response to DNA-damage *GADD* genes induce arrest in cell cycle progression at G1/S or G2/M checkpoints.^26^ Hastwell *et al.*^27^ developed an assay that exploits a reporter system in which the expression of EGFP is controlled by regulatory elements of the *GADD45a* gene hosted in the p53-competent human lymphoblastoid TK6 cell line. A thorough validation of this assay showed its high sensitivity and specificity.^28^ The assay is commercially available as GreenScreen HC assay provided by Gentronics Ltd (UK). Recently Zhang *et al*.^29^ developed a stably transfected HepG2 cell line containing *GADD153* promoter regions coupled to the luciferase reporter gene.

*p21* belongs to p53 mediated DNA damage responsive genes that has not been previously used as an indicator of genotoxic injury. For the construction of our reporter system, we selected the *p21* promoter to drive EGFP expression since recently Ellinger-Ziegelbaure *et al.* 17 reported that *p21* was up-regulated only by genotoxic carcinogens in the liver of rats exposed to genotoxic and non-genotoxic carcinogens. The *GADD45a* gene was up-regulated by both, genotoxic and non-genotoxic carcinogens. Therefore, it could be that our test system will allow for discrimination of the two types of carcinogens.

We evaluated the sensitivity of the assay and established optimal exposure conditions for induced EGFP fluorescence data collection using four model genotoxic agents with known mechanisms of action. The results showed that the optimal exposure time for detection of EGFP expression is 48 h. Although the EGFP fluorescence in cells exposed to MMS, BaP and CisPt increased with the time of exposure, the lowest effective concentration (LOEC) at which a significant increase in EGFP fluorescence was observed did not change. Longer exposures lead to reduced cell viability, resulting either from cytotoxicity or inhibition of cell division that may interfere with the reliability of EGFP fluorescence detection and calculation of the relative EGFP induction ratio as a quantitative measure of genotoxic activity. When measurements of EGFP fluorescence are performed in wells with a very different number of control vs. treated cells, interference with the optical measurements due to changes in the background reflectance and absorbance of the microplate is possible. The half-life of EGFP in mammalian cells has been reported to be in the range of 24 – 48 h.^30^,^31^ As the relative EGFP induction ratio is normalized to the cell viability, which was significantly reduced after prolonged exposure, normalization might give unreliable high values of the EGFP induction ratio due to the accumulated EGFP. The reason for unreliable results can also be cytotoxicity per se. The breakdown of cell integrity can lead to non-specific DNA damage and thus to p21 activation, which does not lead to genetic consequence if cells are dying or dead. Therefore, only the EGFP measurements at which cell viability was not reduced by more than 20% were considered as relevant for genotoxicity evaluation while reduction of cell viability by more than 30% was considered as cytotoxic.

The alkylating agent MMS is a known mutagen and rodent carcinogen.^32^,^33^ Recently, it has been reported that MMS induces phosphorylation of the p53 protein and increases its DNA-binding properties to cause an increased expression of *p21*.^34^ MMS induced a dose- dependent increase of EGFP fluorescence with a LOEC of 20 μg/mL. The sensitivity of our system for MMS genotoxicity detection is similar to that of the GreenScreen HC assay with the *GADD45a* promoter fused to an EGFP gene, in which the LOEC was 25 μg/mL^27^, and to that with the *p53R2* promoter fused to the luciferase reporter in MCF-7 cells in which the LOEC was around 10 μg/mL.^25^

BaP is an indirectly-acting genotoxic carcinogen that is metabolized by cytochrome P450 enzymes to diol epoxide BPDE, which binds covalently to guanine bases.^35^ Exposure to BaP is known to induce activation of the p53 protein and its downstream regulated genes including *p21*.^36^,^37^ The LOEC for BaP was at 0.13 μg/mL (0.5 μM), and at the highest tested concentration 1.26 μg/mL (5 μM) the relative EGFP induction ratio was 8.54 after 24 h exposure. HepG2 cells transfected with GADD153 fused to luciferase were significantly more sensitive for BaP genotoxicity detection; the LOEC was 0.0025 μg/mL (10 nM).^29^ The authors ascribed high sensitivity of their assay compared to other reporter systems to the sensitivity of luciferase, which seems to be higher than that of EGFP.^29^ In MCF-7 cells transfected with *p532R* coupled to the luciferase reporter gene, the LOEC for BaP was 0.26 μg/mL when tested without metabolic activation and 0.12 μg/mL in the presence of metabolic activation.^24^ The lower sensitivity of MCF-7 cells in the absence of metabolic activation compared to HepG2 cells can be ascribed to their lower expression of metabolic enzymes. When using metabolically incompetent cells, the indirectly-acting genotoxic agents have to be tested in the presence of exogenous metabolic activation, usually S9 liver extracts. However, S9 is light-absorbing and fluorescent that can confound spectrophotometric measurements of fluorescence, which is the main limitation of reporter systems based on EGFP. For the GreenScreen HC test system, a protocol based on flow cytometry (FCM) has been developed for the detection of indirectly-acting genotoxic chemicals, and the LOEC for BaP was 1.25 μg/mL.^38^ Thus, our test system with HepG2 cells represents great potential for direct detection of the indirectly-acting genotoxic agents.

A DNA cross-linker CisPt induces bulky lesions, which block DNA transcription *in vitro*.^39^ The response to CisPt-induced DNA damage activates p53 through the ATR-Chk2 pathway.^40^ The bulky DNA damage induced by different genotoxic chemicals such as DNA cross-linkers or BaP are repaired by nucleotide excision repair (NER). The studies showed that triggering of the signal transduction cascade that leads to phosphorylation of p53 or Chk1 requires recognition and processing of the lesions by NER.^41^ In p21HepG2GFP, CisPt induced a dose-dependent induction of EGFP fluorescence. The LOEC was 0.41 μg/mL, which is more sensitive compared to the response observed with the GreenScreen HC assay in which the LOEC was 1 μg/mL.^27^ The MCF-7 cells carrying the *p53R2* promoter linked to the luciferase reporter were less sensitive; the LOEC was around 10 μg/mL.^25^

VBL belongs to spindle poisons that block polymerization of tubulin into microtubules and inhibit cell division without directly damaging DNA.^42^ These chemicals induce activation of p53 and cell cycle arrest mediated by p21^43^, although the details of this process are not clear. VBL induced a significant increase of EGFP fluorescence at the lowest tested concentration of 0.1 μg/mL, which decreased at higher concentrations. VBL showed a cytostatic effect, which is reflected in rapid decrease of relative cell viability during prolonged exposure. At all tested concentrations, the relative cell viability was reduced by 20% or more already after 48 h exposure. Therefore, only the effect observed after 24 h exposure was considered. Lower induction of *p21*-mediated EGFP expression at higher concentrations may be explained by its toxicity. In MCF-7 cells with the *p53R2*-mediated luciferase reporter, VBL induced comparable cytotoxicity and induction of the reporter gene^25^ as we observed in our test system. VBL was highly cytotoxic also in the GreenScreen HC test with LOEC for growth inhibition and GFP induction at 0.02 μg/mL.^27^

In conclusion, our study showed that the new biosensor system with the human hepatoma cell line p21HepG2GFP efficiently detects different types of genotoxic agents. Its main advantages are the use of metabolically competent human cells that allow for direct detection of indirectly-acting genotoxic chemicals and spectrofluorimetric measurement of reporter genes on a microplate format ensuring easy handling and rapid data acquisition. After further validation of the test system, which is currently in progress, this genotoxicity assay based on *p21* gene expression can become a valuable tool with potential applications in the fields of chemical and drug safety evaluation as well as for environmental and occupational monitoring of exposure to chemical agents.

## Figures and Tables

**TABLE 1:** Cell viability and induction of EGFP fluorescence in p21 HepG2 GFP cells exposed to Methylmethane sulphonate (MMS), Benzo[a]pyrene (BaP), Cisplatin (CisPt) and Vinblastine (VLB) for 24, 48, 72, 120 and 168 h.

**MMS**	**24 hours**			**48 hours**			**72 hours**			**120 hours**			**168 hours**		
Conc. μg/ml	Viab. (%) ± SD^a^	GFP int. ± SD^b^	GFP ind. ± SD^c^	Viab. (%) ± SD^a^	GFP int. ± SD^b^	GFP ind. ± SD^c^	Viab. (%) ± SD^a^	GFP int. ± SD^b^	GFP ind. ± SD^c^	Viab. (%) ± SD^a^	GFP int. ± SD^b^	GFP ind. ± SD^c^	Viab. (%) ± SD^a^	GFP int. ± SD^b^	GFP ind. ± SD^c^
**0**	100 ± 0,05	8,8 ± 0,87	1,00 ± 0,10	100 ± 0,02	12,8 ± 1,29	1,00 ± 0,10	100 ± 0,03	15,4 ± 1,58	1,00 ± 0,10	100 ± 0,06	10,5 ± 1,42	1,00 ± 0,14	100 ± 0,06	13,4 ± 1,94	1,00 ± 0,14
**5,00**	102 ± 0,04	9,4 ± 0,98	1,04 ± 0,10	117 ± 0,04	13,8 ± 1,79	0,92 ± 0,12	91 ± 0,04	16,1 ± 1,34	1,14 ± 0,09	79 ± 0,06	12,9 ± 1,03	1,56 ± 0,12	67 ± 0,08	16,7 ± 1,11	1,85 ± 0,11
**10,00**	108 ± 0,03	10,6 ± 1,17	1,11 ± 0,12	106 ± 0,02	15,8 ± 1,48	1,16 ± 0,11	104 ± 0,03	18,7 ± 2,08	1,16 ± 0,12	87 ± 0,05	18,5 ± 2,16	2,02 ± 0,17	63 ± 0,05	20,6 ± 1,13	2,43 ± 0,11
**20,00**	104 ± 0,04	10,8 ± 1,06	1,17 ± 0,11	109 ± 0,02	18,6 ± 1,47	1,33 ± 0,11	107 ± 0,03	22,6 ± 1,58	1,37 ± 0,10	79 ± 0,06	23,3 ± 0,87	2,82 ± 0,11	60 ± 0,06	25,1 ± 2,47	3,13 ± 0,16
**40,00**	110 ± 0,04	10,6 ± 1,13	1,09 ± 0,11	110 ± 0,02	20,5 ± 1,07	1,45 ± 0,09	102 ± 0,04	26,3 ± 1,58	1,67 ± 0,10	59 ± 0,05	24,8 ± 1,56	4,04 ± 0,14	37 ± 0,05	25,3 ± 2,17	5,03 ± 0,15
**50,00**	88 ± 0,04	10,4 ± 0,75	1,33 ± 0,09	104 ± 0,02	20,1 ± 1,35	1,51 ± 0,10	94 ± 0,03	25,5 ± 1,78	1,76 ± 0,11	51 ± 0,04	23,4 ± 0,81	4,34 ± 0,11	27 ± 0,04	24,5 ± 0,81	6,74 ± 0,10

**BaP**	**24 hours**			**48 hours**			**72 hours**			**120 hours**			**168 hours**		
Conc. μg/ml	Viab. (%) ± SD^a^	GFP int. ± SD^b^	GFP ind. ± SD^c^	Viab. (%) ± SD^a^	GFP int. ± SD^b^	GFP ind. ± SD^c^	Viab. (%) ± SD^a^	GFP int. ± SD^b^	GFP ind. ± SD^c^	Viab. (%) ± SD^a^	GFP int. ± SD^b^	GFP ind. ± SD^c^	Viab. (%) ± SD^a^	GFP int. ± SD^b^	GFP ind. ± SD^c^
**0**	100 ± 0,05	9,8 ± 1,12	1,00 ± 0,11	100 ± 0,01	14,3 ± 0,98	1,00 ± 0,07	100 ± 0,08	17,6 ± 1,60	1,00 ± 0,09	100 ± 0,04	13,0 ± 1,30	1,00 ± 0,10	100 ± 0,04	17,1 ± 1,60	1,00 ± 0,09
**0,05**	110 ± 0,04	12,5 ± 1,42	1,16 ± 0,13	100 ± 0,02	15,5 ± 1,38	1,08 ± 0,08	91 ± 0,12	19,1 ± 1,87	1,19 ± 0,10	66 ± 0,04	16,2 ± 2,06	1,89 ± 0,13	55 ± 0,05	22,2 ± 1,77	2,36 ± 0,10
**0,13**	119 ± 0,03	18,1 ± 1,06	1,55 ± 0,11	109 ± 0,01	20,7 ± 1,73	1,33 ± 0,09	99 ± 0,11	24,7 ± 2,40	1,42 ± 0,11	58 ± 0,05	21,0 ± 1,30	2,79 ± 0,10	32 ± 0,04	26,2 ± 1,69	4,79 ± 0,10
**0,25**	112 ± 0,04	23,8 ± 1,04	2,17 ± 0,11	104 ± 0,02	27,1 ± 1,88	1,82 ± 0,10	98 ± 0,10	31,2 ± 2,50	1,81 ± 0,12	50 ± 0,03	28,0 ± 1,15	4,31 ± 0,09	23 ± 0,03	33,6 ± 0,56	8,54 ± 0,06
**0,50**	101 ± 0,04	39,9 ± 1,46	4,03 ± 0,13	105 ± 0,02	41,3 ± 2,13	2,75 ± 0,11	84 ± 0,08	45,4 ± 2,38	3,07 ± 0,11	46 ± 0,03	43,0 ± 1,50	7,19 ± 0,11	20 ± 0,03	45,7 ± 2,98	13,36 ± 0,13
**1,26**	96 ± 0,04	80,3 ± 2,55	8,54 ± 0,19	99 ± 0,02	90,8 ± 3,18	6,41 ± 0,14	76 ± 0,11	91,4 ± 4,14	6,83 ± 0,16	41 ± 0,03	80,0 ± 4,39	15,01 ± 0,22	17 ± 0,02	79,3 ± 2,01	27,28 ± 0,11

**CisPt**	**24 hours**			**48 hours**			**72 hours**			**120 hours**			**168 hours**		
Conc. μg/ml	Viab. (%) ± SD^a^	GFP int. ± SD^b^	GFP ind. ± SD^c^	Viab. (%) ± SD^a^	GFP int. ± SD^b^	GFP ind. ± SD^c^	Viab. (%) ± SD^a^	GFP int. ± SD^b^	GFP ind. ± SD^c^	Viab. (%) ± SD^a^	GFP int. ± SD^b^	GFP ind. ± SD^c^	Viab. (%) ± SD^a^	GFP int. ± SD^b^	GFP ind. ± SD^c^
**0**	100 ± 0,05	13,5 ± 1,50	1,00 ± 0,11	100 ± 0,02	21,0 ± 1,89	1,00 ± 0,09	100 ± 0,08	24,1 ± 1,25	1,00 ± 0,05	100 ± 0,01	16,4 ± 2,26	1,00 ± 0,04	100 ± 0,11	11,3 ± 0,97	1,00 ± 0,02
**0,41**	90 ± 0,04	17,8 ± 2,28	1,47 ± 0,14	98 ± 0,02	25,4 ± 2,75	1,23 ± 0,11	92 ± 0,07	33,5 ± 3,48	1,51 ± 0,10	70 ± 0,01	26,8 ± 2,63	2,33 ± 0,05	41 ± 0,08	18,6 ± 0,79	4,01 ± 0,02
**0,83**	103 ± 0,04	17,6 ± 1,55	1,27 ± 0,11	97 ± 0,02	27,2 ± 2,56	1,34 ± 0,11	102 ± 0,08	36,3 ± 2,87	1,48 ± 0,09	71 ± 0,01	30,8 ± 2,46	2,65 ± 0,04	37 ± 0,06	20,6 ± 0,59	4,93 ± 0,02
**1,65**	105 ± 0,05	18,6 ± 1,85	1,31 ± 0,12	89 ± 0,02	35,6 ± 2,26	1,90 ± 0,10	89 ± 0,07	48,8 ± 2,59	2,28 ± 0,08	56 ± 0,01	38,2 ± 1,34	4,16 ± 0,03	27 ± 0,06	25,7 ± 0,90	8,42 ± 0,02
**3,30**	100 ± 0,05	18,9 ± 1,57	1,40 ± 0,11	71 ± 0,02	39,8 ± 1,83	2,67 ± 0,09	80 ± 0,09	54,5 ± 2,48	2,83 ± 0,08	34 ± 0,01	41,5 ± 1,23	7,44 ± 0,03	22 ± 0,06	31,7 ± 0,85	12,75 ± 0,02
**6,60**	96 ± 0,05	20,0 ± 1,63	1,54 ± 0,12	51 ± 0,02	33,7 ± 3,35	3,15 ± 0,12	48 ± 0,09	43,2 ± 3,97	3,73 ± 0,11	21 ± 0,02	38,2 ± 2,43	11,09 ± 0,04	14 ± 0,07	32,8 ± 1,90	20,73 ± 0,03

**VLB**	**24 hours**			**48 hours**			**72 hours**			**120 hours**			**168 hours**		
Conc. μg/ml	Viab. (%) ± SD^a^	GFP int. ± SD^b^	GFP ind. ± SD^c^	Viab. (%) ± SD^a^	GFP int. ± SD^b^	GFP ind. ± SD^c^	Viab. (%) ± SD^a^	GFP int. ± SD^b^	GFP ind. ± SD^c^	Viab. (%) ± SD^a^	GFP int. ± SD^b^	GFP ind. ± SD^c^	Viab. (%) ± SD^a^	GFP int. ± SD^b^	GFP ind. ± SD^c^
**0**	100 ± 0,01	13,0 ± 0,45	1,00 ± 0,03	100 ± 0,00	18,2 ± 0,78	1,00 ± 0,04	100 ± 0,04	20,8 ± 1,27	1,00 ± 0,06	100 ± 0,03	18,5 ± 1,06	1,00 ± 0,06	100 ± 0,10	20,8 ± 1,17	1,00 ± 0,06
**0,10**	94 ± 0,01	26,3 ± 1,89	2,15 ± 0,09	81 ± 0,01	25,9 ± 2,01	1,76 ± 0,08	57 ± 0,01	28,7 ± 1,50	2,41 ± 0,07	18 ± 0,02	18,2 ± 1,12	5,46 ± 0,06	5 ± 0,01	17,2 ± 1,22	16,55 ± 0,05
**0,50**	95 ± 0,01	24,3 ± 2,87	1,96 ± 0,13	80 ± 0,01	22,3 ± 2,27	1,54 ± 0,08	58 ± 0,01	20,7 ± 1,50	1,71 ± 0,07	14 ± 0,00	12,2 ± 0,92	4,70 ± 0,05	4 ± 0,00	11,2 ± 1,22	13,45 ± 0,05
**1,00**	90 ± 0,00	19,6 ± 0,49	1,67 ± 0,04	85 ± 0,00	18,0 ± 0,75	1,17 ± 0,04	56 ± 0,01	14,9 ± 0,58	1,28 ± 0,04	16 ± 0,02	9,6 ± 1,01	3,24 ± 0,06	5 ± 0,01	8,3 ± 0,57	8,03 ± 0,04
**2,50**	85 ± 0,01	17,2 ± 0,82	1,55 ± 0,05	77 ± 0,01	15,0 ± 1,08	1,07 ± 0,05	52 ± 0,00	11,7 ± 0,47	1,08 ± 0,04	16 ± 0,01	7,3 ± 0,78	2,45 ± 0,05	5 ± 0,01	7,3 ± 0,80	7,07 ± 0,05
**5,00**	78 ± 0,04	10,4 ± 0,76	1,03 ± 0,05	70 ± 0,01	7,9 ± 1,07	0,62 ± 0,05	42 ± 0,01	9,6 ± 0,75	1,10 ± 0,05	15 ± 0,00	3,3 ± 0,56	1,17 ± 0,04	6 ± 0,01	3,7 ± 0,67	2,95 ± 0,04

a Cell viability was measured with the MTS assay and is expressed as % of viable p21 HepG2 GFP cells treated with MMS, BaP, CisPt and VLB compared to control, non-treated cells.

b Intensity of EGFP fluorescence measured at 485 nm excitation and 535 nm emission wavelengths.

c Relative EGFP induction expressed as the ratio between the EGFP fluorescence intensity of the treated cells and non-treted control cells, normalized to cell viability.

Light grey areas represent significantly different values compared to control (P<0.001)

Dark grey areas represent values of cell viability below 70% of control.

## References

[1] Elespuru RK, Agarwal R, Atrakchi AH, Bigger CAH, Heflich RH, Jagannath DR (2009). Current and Future Application of Genetic Toxicity Assays: The Role and Value of In Vitro Mammalian Assays. Toxicol Sci.

[2] Sutton MD, Smith BT, Godoy VG, Walker GC (2000). The SOS response: Recent insights into umuDC-dependent mutagenesis and DNA damage tolerance. Ann Rev Genet.

[3] Putnam CD, Jaehnig EJ, Kolodner RD (2009). Perspectives on the DNA damage and replication checkpoint responses in Saccharomyces cerevisiae. DNA Repair.

[4] Holbrook NJ, Fornace AJ (1991). Response to adversity - molecular control of gene activation following genotoxic stress. New Biologist.

[5] Quillardet P, Huisman O, Dari R, Hofnung M (1982). SOS chromotest, a direct assay of induction of an sos function in escherichia-coli k-12 to measure genotoxicity. P Natl Acad Sci USA.

[6] Oda Y, Nakamura S, Oki I, Kato T, Shinagawa H (1985). Evaluation of the new system (umu-test) for the detection of environmental mutagens and carcinogens. Mutat Res.

[7] Walmsley RM, Billinton N, Walsh L, Barker MG, Knight AW, Cahill PA (2003). A yeast RAD54-GFP genotoxicity assay, is effective in identifying direct acting mutagens in addition to clastogens not detected by bacterial tests. Toxicol Sci.

[8] Liu X, Kramer JA, Swaffield JC, Hu Y, Chai G, Wilson AGE (2008). Development of a highthroughput yeast-based assay for detection of metabolically activated genotoxins. Mutat Res-Gen Tox En.

[9] Zhou B-BS, Elledge SJ (2000). The DNA damage response: putting checkpoints in perspective. Nature.

[10] Sionov RV, Haupt Y (1999). The cellular response to p53: the decision between life and death. Oncogene.

[11] Waldman T, Kinzler KW, Vogelstein B (1995). P21 is necessary for the P53-mediated G1 arrest in human cancer cells. Cancer Res.

[12] Vogelstein B, Lane D, Levine AJ (2000). Surfing the p53 network. Nature.

[13] Moldovan G-L, Pfander B, Jentsch S (2007). PCNA, the maestro of the replication fork. Cell.

[14] Park SY, Lee SM, Ye SK, Yoon SH, Chung MH, Choi J (2006). Benzo[a]pyrene-induced DNA damage and p53 modulation in human hepatoma HepG2 cells for the identification of potential biomarkers for PAH monitoring and risk assessment. Toxicol Lett.

[15] Zegura B, Zajc I, Lah TT, Filipic M (2008). Patterns of microcystin-LR induced alteration of the expression of genes involved in response to DNA damage and apoptosis. Toxicon.

[16] Hreljac I, Zajc I, Lah T, Filipic M (2008). Effects of model organophosphorous pesticides on DNA damage and proliferation of HepG2 cells. Environ Mol Mutagen.

[17] Ellinger-Ziegelbauer H, Stuart B, Wahle B, Bomann W, Ahr HJ (2005). Comparison of the expression profiles induced by genotoxic and nongenotoxic carcinogens in rat liver. Mutat Res-Gen Tox En.

[18] Knasmuller S, Mersch-Sundermann V, Kevekordes S, Darroudi F, Huber WW, Hoelzl C (2004). Use of human-derived liver cell lines for the detection of environmental and dietary genotoxicants; current state of knowledge. Toxicology.

[19] Bressac B, Galvin KM, Liang TJ, Isselbacher KJ, Wands JR, Ozturk M (1990). Abnormal structure and expression of p53 gene in human hepatocellular-carcinoma. P Natl Acad Sci USA.

[20] Mesojednik S, Kamensek U, Cemazar M (2008). Evaluation of shRNA-mediated gene silencing by electroporation in LPB fibrosarcoma cells. Radiol Oncol.

[21] Todd MD, Lee MJ, Williams JL, Nalezny JM, Gee P, Benjamin MB (1995). The cat-tox (l) assay - a sensitive and specific measure of stress-induced transcription in transformed human liver-cells. Fund Appl Toxicol.

[22] Tanaka H, Arakawa H, Yamaguchi T, Shiraishi K, Fukuda S, Matsui K (2000). A ribonucleotide reductase gene involved in a p53-dependent cell-cycle checkpoint for DNA damage. Nature.

[23] Guittet O, Hakansson P, Voevodskaya N, Fridd S, Graslund A, Arakawa H (2001). Mammalian p53R2 protein forms an active ribonucleotide reductase in vitro with the R1 protein, which is expressed both in resting cells in response to DNA damage and in proliferating cells. J Biol Chem.

[24] Ohno K, Tanaka-Azuma Y, Yoneda Y, Yamada T (2005). Genotoxicity test system based on p53R2 gene expression in human cells: Examination with 80 chemicals. Mutat Res-Gen Tox En.

[25] Ohno K, Ishihata K, Tanaka-Azuma Y, Yamada T (2008). A genotoxicity test system based on p53R2 gene expression in human cells: Assessment of its reactivity to various classes of genotoxic chemicals. Mutat Res-Gen Tox En.

[26] Siafakas RA, Richardson DR (2009). Growth arrest and DNA damage-45 alpha (GADD45α). Int J BiochemCell B.

[27] Hastwell PW, Chai LL, Roberts KJ, Webster TW, Harvey JS, Rees RW (2006). High-specificity and high-sensitivity genotoxicity assessment in a human cell line: Validation of the GreenScreen HC GADD45a-GFP genotoxicity assay. Mutat Res-Gen Tox En.

[28] Birrell L, Cahill P, Hughes C, Tate M, Walmsley RM (2010). GADD45a-GFP GreenScreen HC assay results for the ECVAM recommended lists of genotoxic and non-genotoxic chemicals for assessment of new genotoxicity tests. Mutat Res-Gen Tox En.

[29] Zhang R, Niu YJ, Do HR, Cao XW, Shi D, Hao QL (2009). A stable and sensitive testing system for potential carcinogens based on DNA damage-induced gene expression in human HepG2 cell. Toxicol In Vitro.

[30] Yang TT, Cheng LZ, Kain SR (1996). Optimized codon usage and chromophore mutations provide enhanced sensitivity with the green fluorescent protein. Nucleic Acids Res.

[31] Cormack BP, Valdivia RH, Falkow S (1996). FACS-optimized mutants of the green fluorescent protein (GFP). Gene.

[32] Lawley PD (1989). Mutagens as carcinogens - development of current concepts. Mutat Res.

[33] Beranek DT (1990). Distribution of methyl and ethyl adducts following alkylation with monofunctional alkylating-agents. Mutat Res.

[34] Jaiswal AS, Narayan S (2002). S(N)2 DNA-alkylating agent-induced phosphorylation of p53 and activation of p21 gene expression. Mutat Res-Fund Mol M.

[35] Perlow RA, Kolbanovskii A, Hingerty BE, Geacintov NE, Broyde S, Scicchitano DA (2002). DNA adducts from a tumorigenic metabolite of benzo[a]pyrene block human RNA polymerase II elongation in a sequence- and stereochemistry-dependent manner. J Mol Biol.

[36] Wang A, Gu J, Judson-Kremer K, Powell KL, Mistry H, Simhambhatla P (2003). Response of human mammary epithelial cells to DNA damage induced by BPDE: involvement of novel regulatory pathways. Carcinogenesis.

[37] Sadikovic B, Rodenhiser DI (2006). Benzopyrene exposure disrupts DNA methylation and growth dynamics in breast cancer cells. Toxicol Appl Pharm.

[38] Jagger C, Tate M, Cahill PA, Hughes C, Knight AW, Billinton N (2009). Assessment of the genotoxicity of S9-generated metabolites using the GreenScreen HC GADD45a-GFP assay. Mutagenesis.

[39] Corda Y, Job C, Anin MF, Leng M, Job D (1993). Spectrum of DNA platinum adduct recognition by prokaryotic and eukaryotic DNA-dependent RNA-polymerases. Biochemistry.

[40] Pabla N, Huang S, Mi QS, Daniel R, Dong Z (2008). ATR-Chk2 signaling in p53 activation and DNA damage response during cisplatin-induced apoptosis. J Biol Chem.

[41] Marini F, Nardo T, Giannattasio M, Minuzzo M, Stefanini M, Plevani P (2006). DNA nucleotide excision repair-dependent signaling to checkpoint activation. P Natl Acad Sci USA.

[42] Owellen RJ, Hartke CA, Dickerson RM, Hains FO (1976). Inhibition of tubulin-microtubule polymerization by drugs of vinca alkaloid class. Cancer Res.

[43] Tishler RB, Lamppu DM, Park S, Price BD (1995). Microtubule-active drugs taxol, vinblastine, and nocodazole increase the levels of transcriptionally active P53. Cancer Res.

